# Systematic generation of *Drosophila* Wnt transgenes enables the characterization of canonical Wnt signaling

**DOI:** 10.1080/19336934.2026.2624185

**Published:** 2026-02-10

**Authors:** Jamie C. Little, Marc Debrunner, Thom de Hoog, Farzad Tadjik, Chiara J. Kehl, George Hausmann, Erich Brunner, Konrad Basler

**Affiliations:** Department of Molecular Life Sciences, University of Zürich, Zürich, Switzerland

## Abstract

A conserved cohort of signalling pathways orchestrate development and adult homoeostasis. Deregulation of these pathways underlies many diseases. A key set of signals is the family of Wnt ligands. Members of this family are conserved, but a clear understanding of the unique and redundant roles is lacking. Previous efforts to study Wnt ligand function in *Drosophila* have been hampered by the difficulty of generating , functional transgenes. To address this, we have created a complete set of synthesized constructs in an insulated expression system, integrated into the same genomic location, enabling reliable gain-of-function analyses across multiple tissues. Distinct phenotypic outcomes were observed, reflecting both shared and unique features of individual ligands. To define the canonicity of Wnt signalling, we monitored canonical targets such as *Dfz3, notum*, and the *‘naked cuticle’* phenotype in developing tissues and the adult gut. Our findings revealed strong evidence of canonical responses from not only *wg*, but also *DWnt6* and *DWnt10* in the embryo, wing disc, larval gut, and adult gut. In addition, *DWnt2, DWnt4*, and *WntD* produced phenotypes distinct from the control with *DWnt2* and *DWnt4*, showing context-dependent evidence of some canonical activity. While previous studies have suggested regulatory features between *wg* and *DWnt6*, our work provides functional evidence that Wg, DWnt6, and DWnt10 each induce expression of canonical signalling reporters *in vivo*. These findings refine our understanding of redundancy and specificity within the *Drosophila* Wnt family and demonstrate that multiple Wnt ligands can act similarly within the canonical pathway depending on tissue context.

## Introduction

Besides being essential for a wide variety of developmental processes, Wnt signalling is involved in a broad range of diseases, including neurodegeneration, metabolic disorders and many cancers, such as colorectal cancer (CRC) [9–12]. CRC is the third most prevalent cancer worldwide and is the fourth most common cause of cancer-related deaths [13]. Aberrant Wnt pathway activity is implicated in over 80% of CRC. Expression 13 of the 19 Wnt genes in the mouse genome are deregulated in CRC progression [1], however the large number of Wnts makes it difficult to determine the contribution of each Wnt to disease onset and progression.

The *Drosophila* genome is simpler, encoding only seven Wnt proteins. Six of these have a well-defined vertebrate homologue (in parentheses). Wingless (Wnt1), DWnt2 (Wnt7), DWnt4 (Wnt9), DWnt 5 (Wnt5), DWnt6 (Wnt6), and DWnt10 (Wnt10). The seventh, WntD, is not conserved in vertebrates [14–16]. Wingless (Wg) is by far the best studied, predominantly in the paradigm of embryonic and larval epithelia (i.e. wing imaginal disc), which were used to delineate the canonical Wnt signalling pathway [17–19].

Despite this extensive work, the diversity of Wnt ligands has made it challenging to disentangle their individual and overlapping roles, particularly in determining when different Wnts act through distinct outputs versus converging on the same signalling pathway(s), thereby limiting our understanding of functional redundancy. A related challenge is to distinguish how Wnts engage in canonical versus non-canonical signalling. Both pathways are essential in *Drosophila* as well as vertebrates [20,53], yet it is still not known which ligands preferentially activate one pathway or the other, nor in which developmental or tissue contexts these distinctions become functionally important [21–26].

Given the strength of its genetic toolkit, *Drosophila melanogaster* provides an excellent system to begin addressing this gap. Our efforts focused on re-designing *Wnt* overexpression constructs, as previous attempts to generate functional *Wnt* transgenes have faced persistent challenges. A major resource for studying Wnt ligand biology was provided by the Herr and Basler 2012 study [14] which used tagged *UAS-Wnt* transgenes to study ligand production, trafficking, and secretion. While these constructs were important for understanding ligand biology, the epitope tags made them less suitable for assessing downstream signalling strength, as tagging could influence ligand processing and receptor interactions. Attempts to generate untagged *UAS-Wnt* fly lines using this standard expression backbone were often unsuccessful, likely due to leaky expression from the minimal *hsp70* promoter causing toxicity during DNA injection and line recovery, preventing the establishment of viable transgenic lines [27,28] (Figure S1).

To mitigate this issue, some studies employed *UAS-FRT-STOP-FRT* flip-out cassettes to restrict *UAS-Wnt* expression [19], but this approach is technically difficult and not always feasible for systematic comparisons across multiple ligands. Other efforts to generate untagged *UAS-Wnt* transgenes [29] relied on random P-element insertions, resulting in position-dependent effects and variable expression strength, which produced phenotypes difficult to compare across fly lines. To address this variability, site-specific integration vectors were developed [3], enabling stable and reproducible transgene expression, and providing a foundation for a more controlled analysis. Still, with this advance, recovering functional untagged *UAS-Wnt* lines remained challenging because basal promoter activity led to poor viability of fly transformants.

To overcome these limitations and enable direct functional comparisons, we acquired an insulated expression vector and developed a systematic strategy for targeted, site-specific integration of untagged *Wnt open reading frames* (*ORFs*), ensuring stable expression and allowing unbiased assessment of downstream signalling outputs. Standard expression vectors for introducing *UAS* controlled transgenes in *Drosophila melanogaster* typically employ five tandem *UAS* repeats, a truncated *hsp70* promoter, and an *SV40* terminator sequence [3,27,30]. To achieve uniform and strong expression while minimizing leaking activity, we utilized an insulated *UAS-backbone* (developed by Thom de Hoog, Brunner lab) using two 430 bp insulator elements derived from Ty3 retrotransposon sequences to stabilize expression and reduce background activation [2,31,32]. These insulator elements reduce variability caused by position effects by blocking the influence of nearby genomic enhancers or silencers, thus reducing unwanted background activation and promoting more comparable expression levels across constructs [27,28,33]. Using this insulated plasmid, we successfully integrated our newly synthesized *Wnt* cDNAs into the *ZH-attP-86Fb* landing site [3], enabling direct and standardized comparisons across lines.

Here we present the generation and validation of a systematic *UAS-Wnt* overexpression resource for the *Drosophila* community. We demonstrate robust expression of all seven Wnt ligands and show how this toolkit can be used to systematically probe canonical or potential non-canonical signalling activities across multiple tissues.

## Results

### Generation and validation of the UAS-Wnts

**Figure 1.**
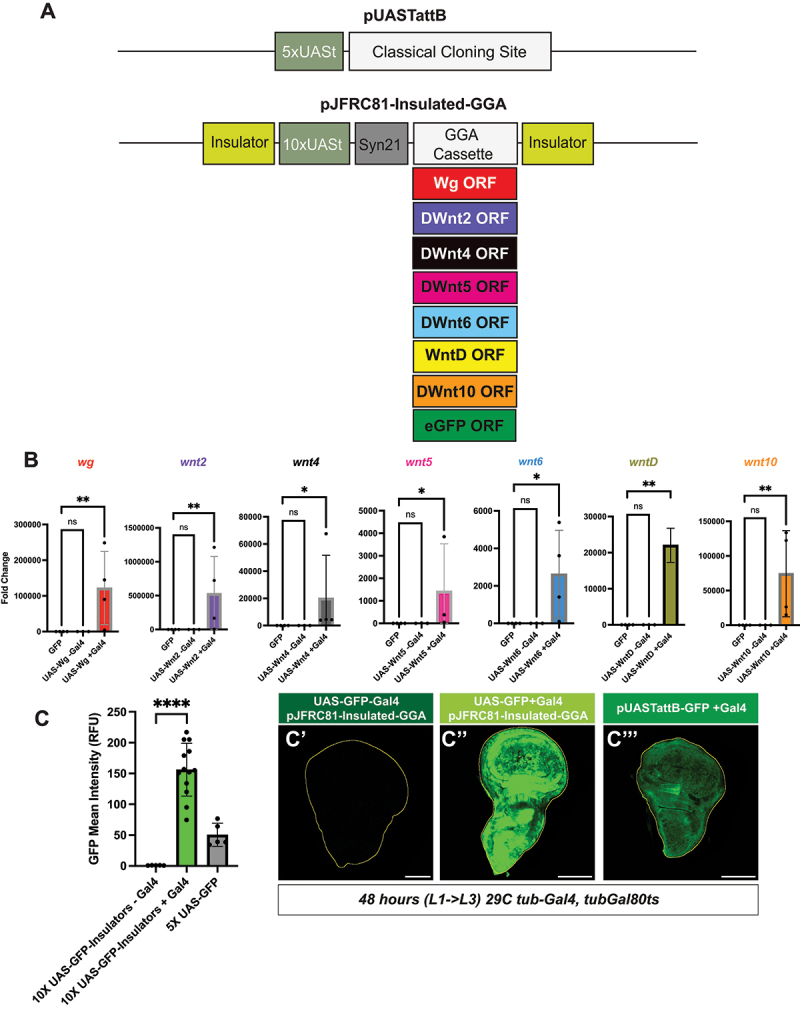
Optimized pJFRC81-Insulated-GGA-Wnt expression system and validation assays. (A) Diagram illustrating modifications to the pUASattB-cDNA plasmid to generate the optimized pJFRC81-Insulated-GGA-Wnt expression construct. The original pUASattB vector contains a 5× UASt sequence (teal green) upstream of a classical cloning site replaced by the desired cDNA (white). The optimized pJFRC81-Insulated-GGA vector incorporates additional features: insulator sequences (lime green) flanking the transgene, a 10× UASt (teal green), the Syn21 translational enhancer element (grey), and an updated cloning cassette (white). (B) Fold change from qRT-PCR analysis of Wnt expression levels in third-instar larvae (N = 3–4 biological replicates) expressing tub-Gal4, tub-Gal80ts and either UAS-GFP, UAS-Wnt (-Gal4), or UAS-Wnt (+Gal4) after 48 h induction. Statistical significance was assessed using one-way ANOVA for wg, DWnt5, DWnt6, WntD, and DWnt10, and Kruskal – Wallis analysis for DWnt2 and DWnt4 (Graphpad PRISM). Significance is indicated as ns (*p* > 0.05), *p < 0.05, **p < 0.01, ***p < 0.001, ****p < 0.0001. For DWnt2, DWnt4, and DWnt5, one biological replicate in the UAS-GFP control group returned an ‘Undetermined’ Ct value, which could not be included in bar graphs or statistical analysis. The absence of detectable signal in controls further supports the observed upregulation of Wnt expression upon induction. (C) GFP relative fluorescence intensity in Drosophila wing imaginal discs, quantified using Imaris (discs outlined in yellow with FIJI). Expression was compared among: (i) UAS-GFP lines carrying pJFRC81-Insulated-GGA without tub-Gal4, tub-Gal80ts induction (48 h), (ii) UAS-GFP with tub-Gal4, tub-Gal80ts induction (48 h), and (iii) UAS-GFP using pUASattB with tub-Gal4, tub-Gal80ts induction (48 h).

Flybase-curated *Wnt cDNA* sequences were used to generate the *UAS*-based expression constructs. The untagged coding sequences (*CDS*) were synthesized *de novo* and cloned into the standard *pUASTattB* vector allowing for targeted integration [3] (Figure 1,Materials and Methods). Standard injection protocols resulted in homozygous viable transgenic flies for *UAS-DWnt5* and *UAS-WntD* in *attP* landing sites *ZH-attp-21F* and *ZH-attp-86Fb* (Fig S1A). Integration of the other *Wnt CDSs* into these sites were not successful: Several thousand embryos (Fig S1A) were injected with varying conditions (25°C, 20°C, 18°C) and concentrations (40ng/uL and 100ng/uL) and invariably resulted in larval or pupal lethality of the injected F0 indicating toxicity due to leaky expression [28]. Given the severe toxicity of some of the *Wnt* transgenes, we decided not to pursue the standard vector option further.

For overcoming this leaky expression, we employed a *UAS*-based plasmid containing flanking insulator elements to stabilize expression [2] (described in detail in the Material and Methods). Genomic insulators generate chromatin loops, thereby shielding regulatory elements such as promoters from interactions with neighbouring enhancers [34]. The resulting modified vector is named pJFRC81-Insulated-GGA (Figure 1). Using this vector, we successfully generated transgenic lines for each Wnt, all of which were either heterozygous or homozygous viable (Fig S1A).

To validate Wnt overexpression in the *UAS-Wnt* lines, we performed quantitative PCR (qPCR). Individual *UAS-Wnt* transgenes were induced using the *tub-Gal4, tubGal80ts* driver system (Figure 1(B), Fig S1B and S1C). Crosses were maintained at 18°C and shifted to 29°C to relieve Gal80 repression and induce transgene expression. RNA was extracted from whole larvae 48 h after induction, corresponding to the transition from early (L2) to third instar (L3) larvae. To ensure accurate comparisons, we used control animals (*UAS-GFP*) that do not overexpress the Wnts. To detect any potential leaky expression, *Wnt* RNA levels were also measured in *UAS-Wnt* animals lacking Gal4. For each Wnt we validated specific *Wnt* primers (Material and Methods: Table 3) and in all cases observed robust and significant upregulation of *Wnt* transcripts in the presence of Gal4 (Figure 1(B), Fig S1B). As an aside, we noted that GFP protein levels from the *UAS-GFP* construct were markedly higher in wing discs than those produced by the commonly used *UAS-GFP* second chromosome line (Figure 1(C)). No GFP signal was detected in control *UAS-GFP* flies lacking the Gal4 driver.

### Embryonic cuticle preparations demonstrate canonical Wnt signalling and aberrant patterning

**Figure 2.**
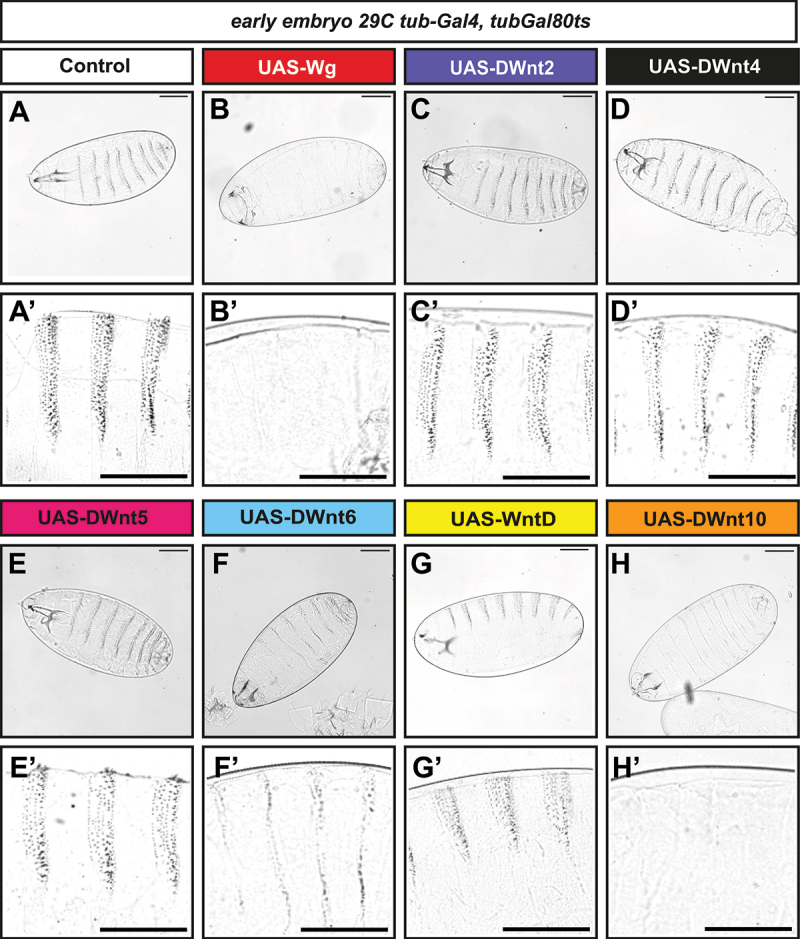
*Cuticle preparations and embryonic segmentation phenotypes upon Wnt overexpression.* (A–H) confocal images of cuticle preparations of *Drosophila* embryos (24 h). Overexpression of (B) *UAS-wg*, (C) *UAS-DWnt6*, and (D) *UAS-DWnt*10 results in a strong *‘naked cuticle’* phenotype characterized by reduced or absent denticle banding, compared with (A) *UAS-GFP* control. Cuticle preparations are presented at 10× magnification (upper panels), with higher-magnification views of *Drosophila* embryos in (A′–H′). Scale bar: 100 µm.

Embryonic cuticle preparations provide a readout of canonical Wnt pathway activation if assayed for the ‘*naked cuticle*’ phenotype [35,36]. As expected, 24 hour expression of gain-of-function *wg* expression produced a robust ‘*naked cuticle*’ phenotype, marked by a near-complete loss of denticle belts and consistent with its established role as a canonical ligand (Figure 2(A)) [4,35]. The expression of *UAS-DWnt6* and *UAS-DWnt10* yielded phenotypes similar to *UAS-wg*, with broad regions absent of denticles, suggesting that these ligands are competent to engage the canonical pathway in the *Drosophila* embryo (Figure 2(F,H)).

In contrast, embryos expressing *DWnt2, DWnt4*, or *WntD* did not display a typical naked phenotype (Figure 2(C,D,G)). Instead, these embryos exhibited variable and often difficult-to-classify cuticle defects, including irregular or patchy denticle patterns that deviated from both control and canonical ‘*naked cuticle*’ phenotypes. These observations suggest that while DWnt2, DWnt4, and WntD can perturb embryonic patterning, their effects are less directly aligned with known canonical signalling output.

### DWnt6 and DWnt10 show canonical reporter activation comparable to wg

To investigate tissue specificity, we used the wing imaginal disc as a classic model for dissecting Wnt signalling, where *wg, DWnt2, DWnt4*, and *DWnt6* are endogenously expressed [37,38]. The canonical target gene, *Dfz3*, serves as a well-established readout of canonical pathway activation in this tissue and others [39–43].

**Figure 3.**
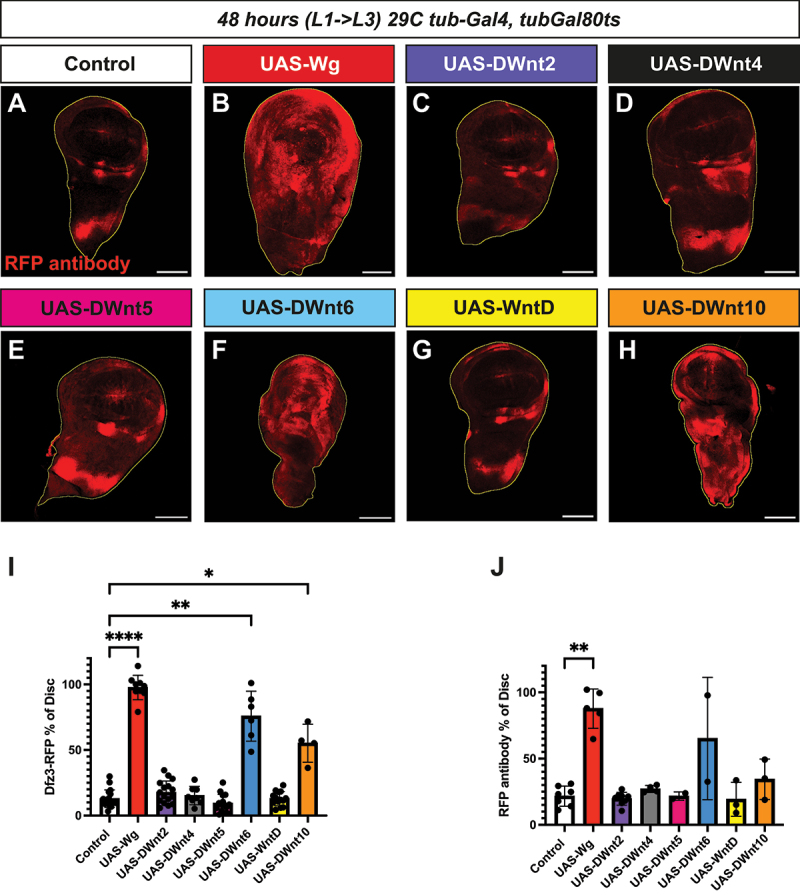
Overexpression of wg, DWnt6, and DWnt10 induces ectopic canonical Wnt signalling in the Drosophila wing disc.

We used the *Gal4/UAS* system in combination with the temperature-sensitive repressor Gal80ts to control the timing of *Wnt* induction. Virgin females carrying *tubGal4, tubGal80ts* were crossed to *UAS-Wnt* males. Progeny were reared at 18°C to suppress Gal4 activity during development, then shifted to 29°C for 48 hours to allow Gal4-dependent expression of the *UAS-Wnt* transgene throughout the larvae. Third instar (L3) larval wing discs were dissected and canonical signalling was monitored by activation of the *Dfz3-RFP* reporter [42,43]. Reporter activity was measured as the proportion of the disc area exhibiting RFP signal relative to the total disc area marked by DAPI staining (Figure 3). Both native RFP fluorescence and RFP antibody staining were assessed; while the native RFP signal was clearly detectable (data not shown), antibody staining produced a more robust and reproducible pattern, with parallel quantifications for both native and antibody-enhanced signals (Figure 3(I,J)).

(A–H) Confocal images of third instar larval wing discs 48 h after induction of the UAS/Gal4 system at 29°C (*tub-Gal4, tub-Gal80ts*). *UAS-transgenes* of the indicated genotypes were expressed together with the canonical Wnt reporter transgene *Dfz3-RFP* (magenta), which marks ectopic Wnt pathway activation. Nuclei were visualized with DAPI, and the overall disc area was segmented and outlined in yellow (Imaris software). *Dfz3-RFP* signal was quantified as a percentage of the total disc area and stained with RFP antibody for visualization (I) Quantification of native RFP-positive area, (J) Quantification of RFP antibody area. Bars indicate mean ± SD. Statistical significance determined by Kruskal–Wallis test (****p < 0.0001, **p < 0.01), *p < 0.05). Scale bar: 100 μm.

In control wing discs, *wg* and *Dfz3-RFP* were expressed in a restricted ring pattern around the pouch and along the dorsal/ventral boundary consistent with previous reports [41]. Overexpression of *wg* using *tub-Gal4, tub-Gal80ts* dramatically expanded this pattern, driving ectopic *Dfz3-RFP* expression throughout the disc (Figure 3(B)). Notably, both *DWnt6* and *DWnt10* elicited similar widespread reporter activation (statistically significant for native RFP) (Figure 3(I)), indicating that these ligands, like Wg, are capable of canonical pathway activation. In contrast, expression of *DWnt2, DWnt4, DWnt5*, nor *WntD* did not alter reporter activity compared to controls (Figure 3(C,D,E,G)).

Together, the classical *Drosophila* embryonic and the larval tissues highlight both the redundancy and specificity in Wnt ligand activity during development, with Wg, DWnt6, and DWnt10 functioning as Wnt ligands capable of inducing canonical reporters, while other ligands show little or no canonical activity under these conditions.

### Overexpression of Wnt ligands in the Drosophila gut reveal distinct canonical activities

In recent years, the *Drosophila* gut has become a focus of many studies, owing to its strong conservation with mammalian systems, particularly in intestinal stem cell maintenance, and its utility as a model for colorectal cancer (CRC), offering an alternative to mammalian studies [44–46]. Given the critical role of Wnt signalling in CRC, we sought to apply our toolkit in this context and highlight its potential for future studies. While the role of Wingless (Wg) in gut development has been well characterized, the contributions of other Wnt ligands remain less understood [47–49]. Transcriptomic studies indicate that the *Wnt* genes are expressed at low levels in the adult gut, but a systematic toolkit for overexpressing and functionally comparing these ligands has not previously been available [50]. The *Drosophila* gut therefore provides a compelling system to assess the canonical activity of our newly synthesized *UAS-Wnt* transgenes.

**Figure 4.**
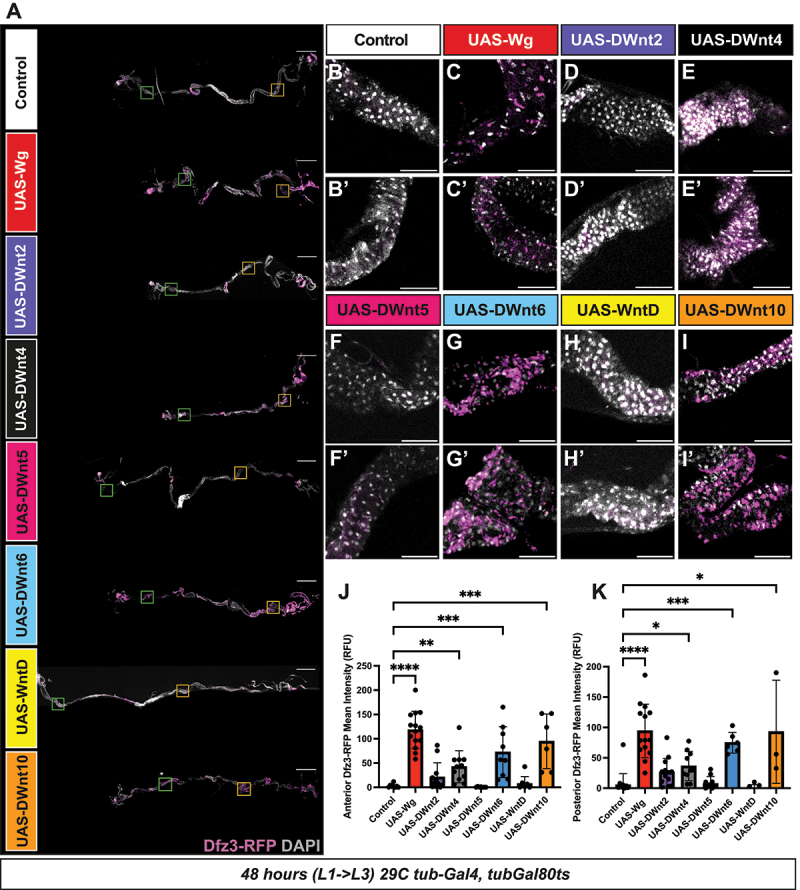
Multiple Wnts promote canonical pathway activation in larval gut progenitors.

In the larval gut, following 48 hours of *wg* induction, we observed robust activation of the canonical reporter *Dfz3-RFP* in adult midgut precursors in both the anterior and posterior regions of the gut (Figure 4(A,C,J,K)). Canonical signalling in larval guts was quantified by measuring nuclear *Dfz3-RFP* intensity following DAPI-based nuclear segmentation within anatomically defined anterior and posterior regions [47], enabling standardized comparisons across genotypes (Figure 4(J-K)). Similar to our previous results in developing tissues, induction of *DWnt6* and *DWnt10* elicited a comparable response, confirming their classification as ligands that can induce a canonical response in these tissues (Figure 4(A,G,I,J,K)). DWnt4 (significantly: *p* < 0.05) and DWnt2 (not significantly: *p* > 0.05) also enhanced *Dfz3-RFP* expression in the anterior and posterior regions (Figure 4(A,D,E,J,K)) though to lesser degree than Wg/DWnt6/DWnt10. By contrast, DWnt5 and WntD did not induce detectable reporter activation, indicating they cannot activate a canonical response (Figure 4(F,H)).

(A) Tiled confocal images (10×) of third instar larval guts 48 h after induction of the UAS/Gal4 system at 29°C (*tub-Gal4, tub-Gal80ts*), expressing the indicated *UAS*-transgenes. Guts were stained with DAPI (grey) and the canonical Wnt reporter *Dfz3-RFP* (magenta). Scale bars: 500 µm. (B–I, B’–I’) Higher-magnification confocal images (green box) of anterior (B – I) and posterior (orange box; B’–I’) gut regions. Adult midgut precursors (AMPs) were identified, and *Dfz3-RFP* signal was quantified using Imaris. Background fluorescence from surrounding non-AMP tissue was measured and subtracted from AMP values. Scale bars: 100 µm. (J, K) Quantification of *Dfz3-RFP* intensity in AMPs (relative fluorescent units, RFU) for anterior (J) and posterior (K) regions after background subtraction using Imaris software. Bars represent mean ± SD. Statistical significance was determined using the Kruskal–Wallis test in Graphpad Prism (****p < 0.0001, ***p < 0.001, **p < 0.01, *p < 0.05).

**Figure 5.**
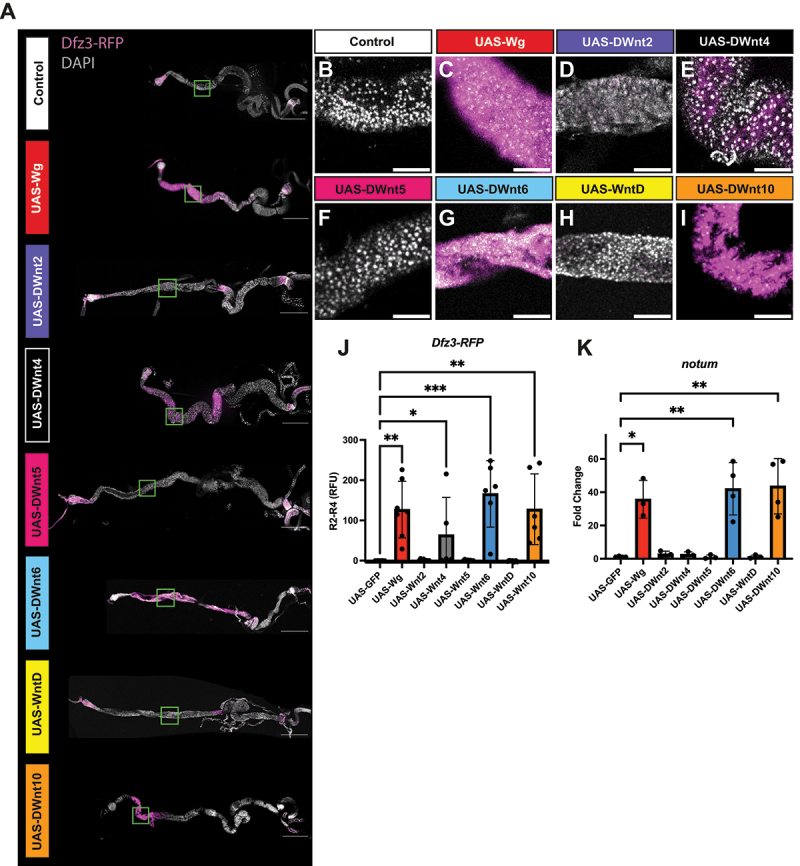
Specific Wnt ligands trigger canonical pathway activation in the anterior adult gut 7 d after induction.

Adult induction was achieved by crossing virgin females carrying *tub-Gal4, tubGal80ts* to *UAS-Wnt* lines. Crosses were reared at 18°C to suppress *Gal4* expression during development; newly eclosed adults (0–12 h) were collected and shifted to 29°C to relieve Gal80ts repression and induce *UAS-Wnt* expression. Midguts were dissected and analysed after 24 h and 7 d at 29°C (Figure 5, Fig S2). In adult midguts *Dfz3-RFP* reporter activity was quantified within defined gut regions, with region boundaries determined by anatomical landmarks and values normalized across samples to allow direct comparison between genotypes.

(A) Tiled confocal images (10×) of 7-day adult guts following induction of the UAS/Gal4 system at 29°C (*tub-Gal4, tub-Gal80ts*), expressing the indicated UAS-transgenes. Guts were stained with DAPI (grey) and the canonical Wnt reporter *Dfz3-RFP* (magenta). Scale bars: 500 µm. (B–I) Higher-magnification confocal images of the R2 anterior gut region (green box in A). Scale bars: 100 µm. (J) Quantification of nuclear *Dfz3-RFP* intensity (relative fluorescent units, RFU) in R2 after background subtraction from R4 using FIJI. Bars represent mean ± SD. (K) qPCR analysis of the canonical Wnt target gene *notum* across all *Wnt* overexpression conditions. Statistical significance was determined using the Kruskal–Wallis test (****p < 0.0001, ***p < 0.001, **p < 0.01, *p < 0.05).

At 24 hours post-induction, ectopic *Dfz3-RFP* reporter activity was observed specifically in region 2 (R2) of guts expressing *wg, DWnt6, and DWnt10*, without obvious morphological changes (Fig S2A, S2C, S2G, S2J and S2I). After 7 d, *Dfz3-RFP* activation in R2 persisted for *wg, DWnt6*, and *DWnt10* (Figure 5(A,C,G,J,I)), and was also detected in *DWnt4* expressing guts (Figure 5(E)). At this later time point, phenotypic changes became more apparent, guts expressing *wg, DWnt6*, and *DWnt4* were noticeably shortened. In contrast, guts expressing *DWnt2, DWnt5*, and *WntD* showed neither ectopic *Dfz3-RFP* expression nor phenotypic changes at either time point.

To complement reporter assays, RNA was extracted from 24 h and 7 day guts for qRT-PCR analysis. This revealed significant (*p* < 0.05) upregulation of *notum*, a canonical Wnt target in the adult gut [47], in guts expressing *wg, DWnt6* and *DWnt10* (Fig S2K, Figure 5(K)).

Together, these experiments demonstrate that under ubiquitous and spatially unrestricted expression using our toolkit, Wg, DWnt6 and DWnt10 reproducibly induce canonical Wnt reporters in the *Drosophila* gut. DWnt4 is also capable of activating canonical reporters but only under prolonged induction and in specific contexts. In contrast, other Wnts either enhance signalling weakly (DWnt2 in larvae) or fail to elicit canonical responses (DWnt5, WntD). These findings identify the gut as a particularly sensitive tissue for dissecting ligand-dependent Wnt responses and highlight the utility of this toolkit for context-dependent analysis of canonical and in future studies, non-canonical outputs across development and disease-relevant contexts.

## Discussion

In this study, we present the first systematic resource for overexpressing the complete set of *Drosophila* Wnt ligands. By employing a *UAS* expression vector containing insulator elements and integrating all constructs into defined genomic landing sites, we overcame longstanding technical barriers that have limited functional analyses of Wnt ligands *in vivo*. Previous attempts to generate Wnt gain-of-function lines frequently resulted in lethality, reflecting the intrinsic potency of Wnt signalling during development. Consistent with this, even within our insulated system we were unable to recover homozygous viable UAS-Wg lines despite extensive chromosome cleaning. This is likely due to low-level basal transgene expression, dosage-dependent effects during development, and tissue-specific sensitivity to elevated Wnt signalling in particular developmental contexts. These constraints highlight the biological potency of Wnt ligands and the technical challenge of developing standardized gain-of-function tools for this pathway.

Using this resource, we clarify the context-dependent capacity of individual Wnt ligands to induce canonical signalling across multiple tissues. Our findings demonstrate how different tissues respond to enforced expression of specific Wnts under standardized gain-of-function conditions. In particular, we identify the gut as a highly sensitive tissue for detecting canonical reporter activation revealing ligand and context-dependent differences in signalling outcomes.

### Context dependent specificity and redundancy among Wnt ligands

Wg, DWnt6, and DWnt10 consistently elicited robust canonical responses across multiple tissues in our experimental settings, DWnt2 and DWnt4 exhibited weaker or delayed canonical activity that was restricted to specific tissues or conditions, consistent with previous reports [29,40,51,52]. Together these findings suggest that multiple Wnts are capable of activating canonical Wnt signalling, but do so selectively in cellular environments that provide the appropriate molecular context.

Building on these observations, DWnt6 and DWnt10 seem to emerge as particularly informative ligands for understanding potential mechanisms of canonical Wnt signalling redundancy. *DWnt6* shares an enhancer with *wg* and displays overlapping expression in the wing disc [7,38,54], while loss of DWnt6 can impair processes typically associated with Wg, suggesting a degree of functional coordination between these ligands [7,8]. Our experiments also reveal a previously underexplored canonical activity for DWnt10. While DWnt10 and DWnt4 have been implicated in non-canonical signalling [51,55], DWnt10 had not been reported to drive canonical signalling, nor to be expressed in the wing disc [37,38]. In our assays, however, DWnt10 produced a striking induction of the ‘*naked cuticle’* phenotype and activated canonical reporters such as *notum* and *Dfz3-RFP* in multiple tissues/contexts. Notably, *DWnt10* resides on chromosome 2 L in the same orientation as *wg* and *DWnt6*, suggesting a shared genomic organization. Together with the known shared enhancer between *wg* and *DWnt6*, these observations also raise the possibility DWnt6 and DWnt10 participate in related regulatory or functional mechanisms. It is conceivable that such mechanisms contribute to buffering or compensation for fluctuations in Wg activity.

Future studies dissecting regulatory interactions and functional interdependence among these ligands will be required to test this hypothesis and to further elucidate how redundancy is organized with the Wnt family in *Drosophila*. Our analyses were performed using a strong, ubiquitous Gal4 driver that induce high levels of ligand expression across all cell types, and therefore reveals signalling competence under maximal, non-physiological conditions. More refined experimental approaches such as using tissue-specific drivers, and varying expression levels will be critical for dissecting how ligand identity, expression level, and cellular context interact to shape canonical versus non-canonical signalling outputs, underscoring the utility of this toolkit for future studies. The availability of our standardized transgenes now enables more direct and controlled examination of these relationships and their downstream signalling outputs.

### Framework for dissecting canonical and non-canonical Wnt signalling outputs

Both shared and distinct features among *Drosophila* Wnt ligands are likely to contribute to their ability to activate canonical signalling. Wg, DWnt6, and DWnt10, share conserved protein domains required for lipid modification and receptor engagement. For example, all three contain a conserved palmitoylation site critical for binding Frizzled receptors and are similar in protein size [56,57]. These structural similarities may explain their ability to activate the canonical pathway through stabilization and nuclear translocation of Armadillo (the *Drosophila* β-catenin homologue), which functions as the transcriptional effector [58–60]. Importantly, this toolkit provides a powerful platform for future studies examining ligand–receptor interactions and downstream signalling mechanisms that confer canonical signalling specificity.

DWnt5, and WntD did not robustly activate canonical reporters, while DWnt4 and to some extent DWnt2, exhibited context dependent weaker canonical responses in some settings. Unlike the canonical pathway, where targets such as *naked, notum*, and *Dfz3* provide direct transcriptional readouts [4–6,47–52], there are currently no established molecular targets for non-canonical Wnt signalling in *Drosophila*. As a result, non-canonical activity has largely been inferred through phenotypic observations, such as changes in polarity, activation of Rho-GTPases, Wnt/PCP dependent asymmetric localization of centrioles/basal bodies, or gastrulation movements, rather than specific transcriptional targets [61–65]. This lack of readily defined readouts represents a major limitation in the field. With the systematic toolkit described here, it should now be possible to go beyond descriptive phenotypes and begin to identify ligand-specific signatures of non-canonical signalling for all Wnts. Thus, in addition to clarifying which ligands induce canonical reporters, our resource opens the door to a more precise dissection of non-canonical pathway specification in *Drosophila.*

### Applications to gut biology and disease models

Beyond developmental biology, these findings have the potential for disease modelling. Aberrant Wnt pathway activation drives many subtypes of CRC and multiple Wnt ligands are deregulated during tumour progression [1]. Recent work by Zipper et al., demonstrates that hormonally regulated, spatially restricted *wg* expression can promote intestinal growth through canonical signalling, as monitored by *fz3* reporters, underscoring the importance of physiological context in shaping Wnt outputs in the gut [66]. In contrast, our study employs strong, ubiquitous overexpression of individual Wnt ligands causing gut shortening, revealing how maximal and spatially unrestrained signalling can elicit distinct outcomes. Together these findings emphasize the highly context-dependent nature of Wnt signalling in the intestine.

By providing a comparative gain-of-function resource, our toolkit enables *Drosophila* gut models to test ligand-specific contributions, explore interactions with APC/β-catenin mutations, and facilitate the identification of potential therapeutic modifiers in a genetically tractable system. Taken together, our work delivers the first complete set of functional *UAS-Wnt* transgenes and establishes a robust framework for evaluating diverse Wnt signalling outputs across tissues. Our findings that DWnt6 and DWnt10 can elicit canonical signalling responses alongside Wg refine current views of redundancy and specificity within the *Drosophila* Wnt family. More broadly, this toolkit may potentially help bridge developmental genetics with disease relevant signalling contexts, creating opportunities to dissect Wnt pathway complexity.

## Materials and methods

### Diagram of the insulated UAS vector

Standard expression vectors such as pUASTattB [3,27,30] typically contain five tandem UAS (teal) elements upstream of a minimal *hsp70* promoter, followed by a multiple cloning site, SV40 *3’UTR* terminator (beige) and a *mini-white* reporter for transgene selection (pink). These constructs are integrated at defined attP landing sites with phiC31 integrase recombination.

For our experiments we used an insulated *UAS-*based vector derived from pJFRC81-10XUAS-IVS-Syn21-GFP-p10 (Addgene #36432) [33], which was kindly provided by Thom de Hoog [2]. This version includes 10x *UAS* repeats (teal), a *Syn21* translational enhancer (grey) and p10 *3’UTR* (beige), flanked by two 430 bp insulator elements derived from Ty3 retrotransposon sequences (bright green) [2,31,32] to stabilize expression and reduce background activation. It also contains a *FRT5* sequence [67], P-element sites P3 (orange)/P5 (cyan) [68], and a SapI flanked CmR-ccdB cassette for complex multi-fragment DNA using GoldenGate Assembly (GGA).

This plasmid is not yet published and was provided by the lab of Damian Brunner. Researchers interested in obtaining the vector may contact Damian Brunner (University of Zürich) directly.

### Cloning of the UAS-transgenes

Wnts cDNAs were synthesized by Genscript based on Flybase-reported reference sequences and cloned into a pUC57 plasmid backbone. The verified inserts were subsequently subcloned into either the stand pUASTattB vector or the insulated UAS-based plasmid described above. (Table 1)

**Table 1.** Cloning Primers.

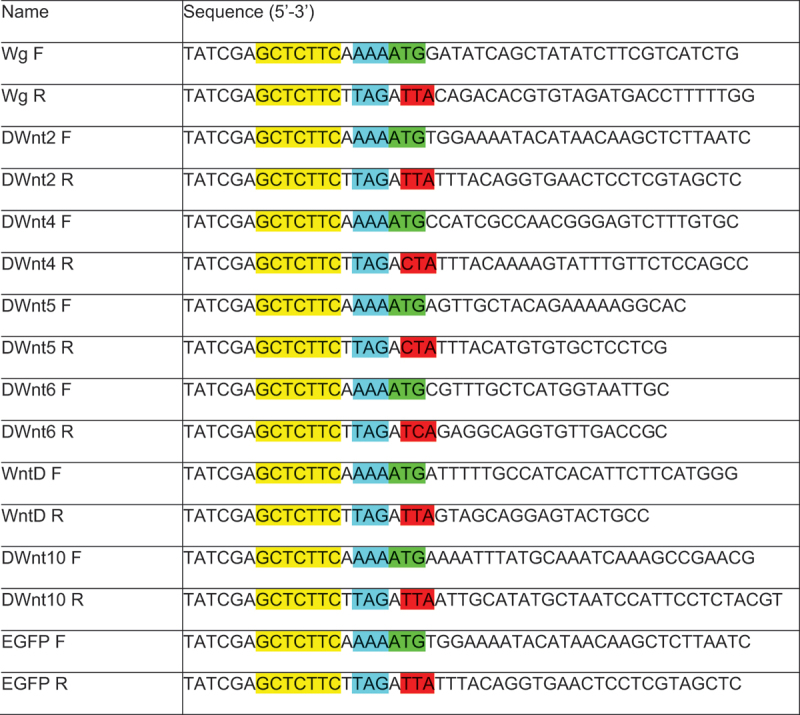

Primer S1 FW: 5’-TATCGAGCTCTTCAAAAATG-cDNA-3’

Primer S5 RV: 5’-TATCGAGCTCTTCTTAGATTA-cDNA-3’


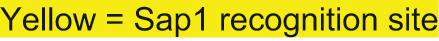



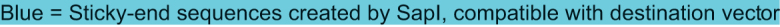



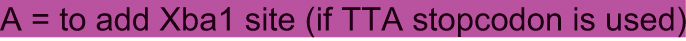



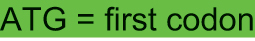



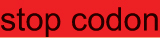


### Drosophila stocks and husbandry

Flies were reared and maintained at 25°C (12:12 h light/dark cycle) on standard corn-meal food containing, per litre, 100 g fresh yeast, 55 g corn meal powder, 10 g organic wheat flour, 8 g agar, 75 g white sugar and 15 ml nipagin. The fly strains used in this study and listed in Table 2. To induce expression of *UAS-Wnt* or *UAS-GFP* transgenes in embryos, larvae, and adult flies, virgin females of the genotype *ywhsFlp; Sp/CyO; tub-Gal4, tub-Gal80ts* were crossed to the respective *UAS*-constructs. Crosses were shifted from 18°C to 29°C at the appropriate developmental stages to achieve temporal control of expression. Transgene induction was assessed across multiple readouts: i) *naked cuticle* phenotypes in embryos (24 h induction), ii) RNA expression in whole larvae (48 h induction), iii) GFP expression in wing discs (48 h induction), iv) canonical target gene induction in wing discs and larval guts (48 h induction), v) canonical target gene induction in adult guts (24 h and 7 d induction).

**Table 2.** Drosophila stocks used in this study.

Genotype	Source
*tub-Gal4* *Chromosome III*	Basler lab
*tub-Gal80ts* *Chromosome III*	BDSC (#7019)
*Sp/cyo; tub-Gal4,tub-Gal80ts* *Chromosome III*	Hugo Stocker
*Tm3, Act-GFP, ser or Tm3,Sb,sqh-mCherry* *(Balancer used for UAS-Wnts* *and tubGal4,tubGal80ts for cuticle preps)* *Chromosome III*	Brunner Lab
*UAS-GFP* *Chromosome II*	Basler lab
*Dfz3-RFP* *Chromosome II*	Kalderon Lab
*yellow*^−^*white*^−^ *Chromosome X*	Basler lab
*UAS-GFP (*pJFRC81-Insulated-GGA) *Chromosome III*	this paper
*UAS-wg (*pJFRC81-Insulated-GGA) *Chromosome III*	this paper
*UAS-DWnt2 (*pJFRC81-Insulated-GGA) *Chromosome III*	this paper
*UAS-DWnt4 (*pJFRC81-Insulated-GGA) *Chromosome III*	this paper
*UAS-DWnt5 (*pJFRC81-Insulated-GGA) *Chromosome III*	this paper
*UAS-DWnt6 (*pJFRC81-Insulated-GGA) *Chromosome III*	this paper
*UAS-WntD (*pJFRC81-Insulated-GGA) *Chromosome III*	this paper
*UAS-DWnt10 (*pJFRC81-Insulated-GGA) *Chromosome III*	this paper

### Immunostaining of imaginal discs and guts

Micro-dissected imaginal discs and gut tissues were fixed in 4% formaldehyde in 1× phosphate-buffered saline (PBS) for 30 min at room temperature (RT), followed by three washes in PBS (10 min each). Samples were then blocked in PBT (1 × PBS containing 0.2% Triton X-100 and 10% HINGS) for 1–2 h at RT. For detection of *Dfz3-RFP* expression, wing discs were incubated with rabbit monoclonal anti-RFP (1:200) at 4°C overnight, washed three times in PBS containing 0.2% Triton X-100 (10 min each), and incubated with Alexa Fluor 647-conjugated secondary antibodies and DAPI (1:1000) for 1 h at RT. For larval and adult guts, as well as GFP-expressing wing discs, tissues were fixed as above, washed three times with 1× PBS, and incubated with DAPI (1:1000) for 1 h at RT. All samples were mounted in Aqua-Polymount (Cat. #18606–20, Polysciences) prior to slide preparation. To minimize tissue compression, guts were mounted Grace Bio-Labs SecureSeal imaging spacer (Cat# GBL654004, Merck/Sigma Aldrich.

### Cuticle preparations

Embryos were dechorinated in 14% bleach for 2 minutes, rinsed with water and mounted in prepared Hoyers medium on a standard microscope slide. Slides were then incubated at 60°C–65°C overnight. Then imaged with brightfield settings (10x) using the Leica Sp8 inverse confocal microscope to observe denticle belts and naked cuticle phenotypes.

### Microscopy and quantifications

Confocal images were acquired using an inverted laser scanning confocal microscope (Leica SP8 Inverse, HC PL APO) equipped with 405 nm (50 mW), 488 nm (20 mW), 552 nm (20 mW) and 638 nm (30 mW) lasers). Embryonic cuticle preps were imaged with 10X/0.3 NA objective while wings discs were imaged with the 20 x/0.3 NA objective. For *Dfz3-RFP* wing disc quantification, total wing disc area (DAPI stained nuclei) and RFP positive regions (native fluorescence and anti-RFP antibody signal) were measured using Imaris software. Larval gut images were acquired at both 10x and 20x magnification. All quantifications were performed using 20x images acquired under identical imaging settings. Because Dfz3-RFP is a nuclear reporter, we segmented nuclei using the DAPI channel and quantified the RFP intensity. Adult midgut progenitors (AMPs) were identified based on their characteristic nuclear morphology (small, clustered nuclei), and background signal was subtracted prior to analysis in Imaris. This approach provides a cell type specific and quantitative readout of canonical Wnt reporter activity in the larval gut. Adult gut images were acquired at 10x magnification. This data was analysed by segmenting nuclei using the DAPI channel and quantifying nuclear Dfz3-RFP in FIJI. Measurements were restricted to defined anatomical regions (regions 2 and 4) [47,69]. We note that adult gut reporter responses under Wnt overexpression conditions were typically binary in nature (on/off). All images were processed and assembled using the Fiji package of ImageJ.

### RNA extraction, cDNA synthesis, and qRT-PCR

Total RNA was extracted from ~10–15 whole third-instar larvae per sample (48 h at 29°C) from the respective genotypes (*n* = 3 independent biological replicates). For adult gut samples, ~15 dissected guts per genotype were collected across three independent experiments. All samples were preserved in RNAlater Stabilization Solution (Thermo Fisher Scientific, Waltham, MA, USA) prior to processing. RNA was isolated using the NucleoSpin RNA Kit (Macherey-Nagel, Cat. 740,955.50, Düren, Germany) according to the manufacturer’s protocol. First-strand cDNA was synthesized using PrimeScript™ RT Master Mix (Takara Bio; see Table 4). Quantitative real-time PCR (qRT-PCR) was performed in technical triplicate with PowerUp SYBR Green Master Mix (Thermo Fisher Scientific; see Table 4) on a QuantStudio 3 Real-Time PCR System (Applied Biosystems). Fold change was calculated by standard qPCR methods.

**Table 3.** qRT-PCR primers used in this study.

Name	Sequence (5’-3’)	Source
wg F	GCAAAATCGTTGATCGAGGCTG	this paper
wg R	CAGGACTCTATCGTTCCTTCACTG	this paper
DWnt2 F	TTGGCGATAGACTCATGCTG	this paper
DWnt2 R	GACGCCTCCAGGTAGATCAG	this paper
DWnt4 F	GCAGCGACGCAAGAAACCC	this paper
DWnt4 R	CTGGCAGTAGTCGCAGATCA	this paper
DWnt5 F	AACGACGAGACCGTATTTGG	this paper
DWnt5 R	GCGAACTTGTAGGCGAACTC	this paper
DWnt6 F	GCACGCTGGTTAAGTTCCAC	this paper
DWnt6 R	CTTCAGCCAGCAGGTCTTCA	this paper
WntD F	ATTCCCCTAGACTCGCTGGT	this paper
WntD R	TGGGATCTCACTCGGTATCC	this paper
DWnt10 F	GAATGGCCCGAAAACTGGAG	this paper
DWnt10 R	CTCGCACTCCACATTACAGC	this paper
Notum F	GCCGGAGAAGCTACAAATAAATG	this paper
Notum R	TCATGCTGGTGTCCCTCAAT	this paper
HK1 (Rpl32F)	AGGCCCAAGATCGTGAAGAA	this paper
HK2 (Rpl32F)	TGTTGCACCAGGAACTTCTTGAA	this paper

**Table 4.** Extended list of materials used in this study.

Name	Manufacturer	Cat. No./ID
Formaldehyde, 16% (w/v), methanol-free	PierceTM	28908
PowerUp SYBR Master Mix	Thermo Fischer	A25742
PrimeScript™ RT Master Mix	TaKaRa	RR036A
Nucleospin RNA	NucleoSpin RNA Kit (Macherey-Nagel, Cat. 740,955.50)	
Aqua-Polymount	Polysciences	Cat. #18606–20,
Grace Bio-Labs SecureSeal imaging spacer	Sigma-Aldrich/Merck, Darmstadt, Germany	Cat. #GBL654004
RNAlater Stabilization Solution	(Thermo Fisher Scientific, Waltham, MA, USA)	

## Supplementary Material

Supplemental Material

## Data Availability

The data that support the findings of this study area available from the corresponding authors (KB), upon reasonable request.
