# Supplementary material for: Systematic generation of Drosophila Wnt transgenes enables the characterization of canonical Wnt signaling
Source: Fly (Austin). 2026 Feb 10;20(1):2624185. doi: 10.1080/19336934.2026.2624185 (PMC12893696; doi:10.1080/19336934.2026.2624185)
Supplement: Supplemental Material [file KFLY_A_2624185_SM1969.docx]

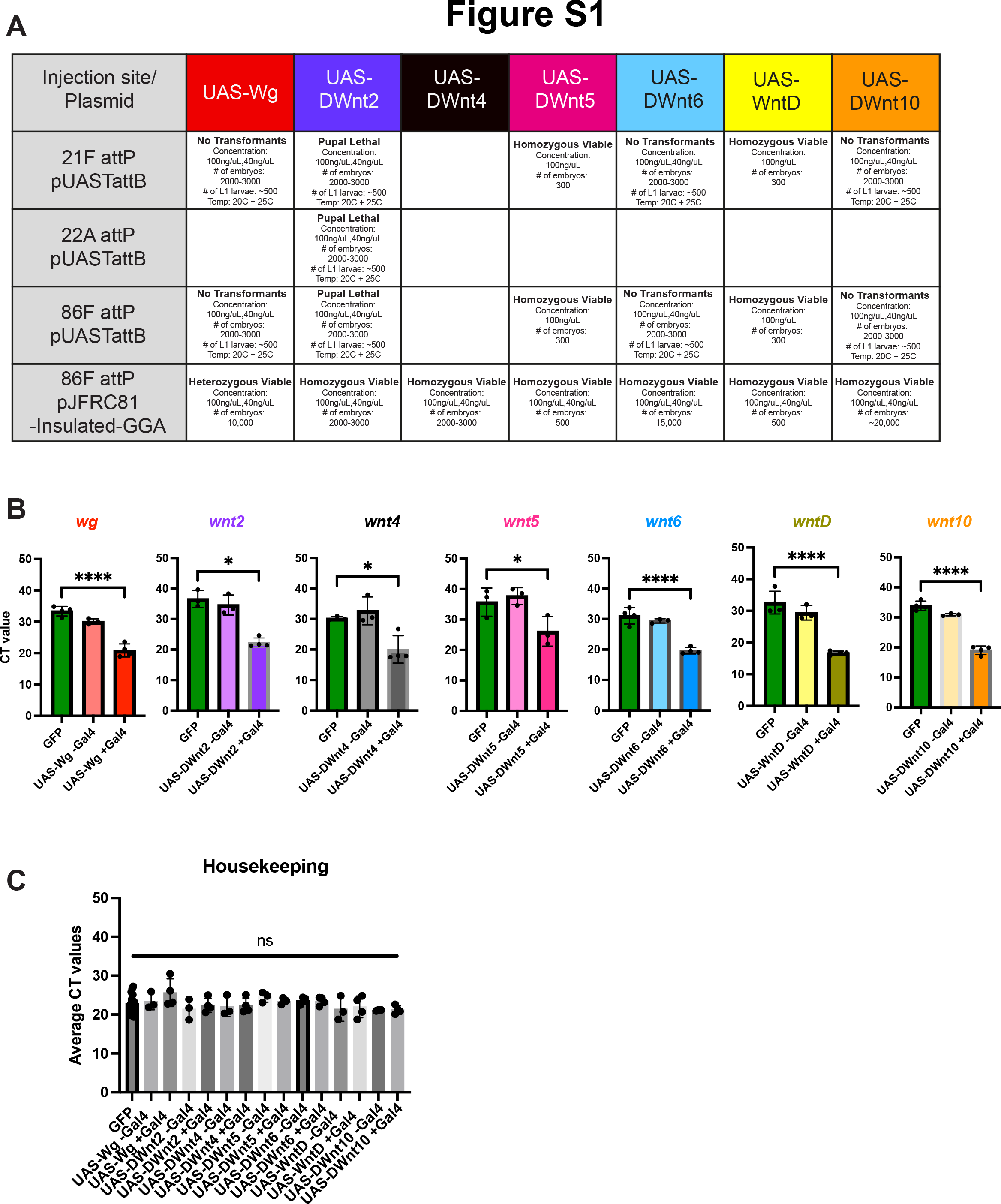


**Fig S1. Transgenic line generation and qRT-PCR analysis of Wnt expression.**
**(A)** Injection outcomes of pUASattB and pJFRC81-Insulated-GGA-Wnt cDNA plasmids at three attP landing sites (*21F, 22A,* and *86F*). The table summarizes injection concentration, number of embryos injected, and resulting viability outcome (homozygous, heterozygous, lethal, or no transformants). **(B–C)** Average Ct values from qRT-PCR analysis of Wnt expression in third-instar larvae carrying tub-Gal4, tub-Gal80ts with either UAS-GFP or *UAS-Wnt* constructs (with or without *Gal4;* induction 48 h at 29 °C). For *DWnt2, DWnt4,* and *DWnt5*, one biological replicate in the *UAS-GFP* control group returned an “Undetermined” Ct value, which could not be included in averages. (B) Average Ct values for Wnt primers under each condition. (C) Summary of average Ct values for housekeeping controls, comparing GFP, −Gal4, and +Gal4 conditions across *Wnt* transgenes. Statistical significance was determined using one-way ANOVA with Graphpad PRISM.


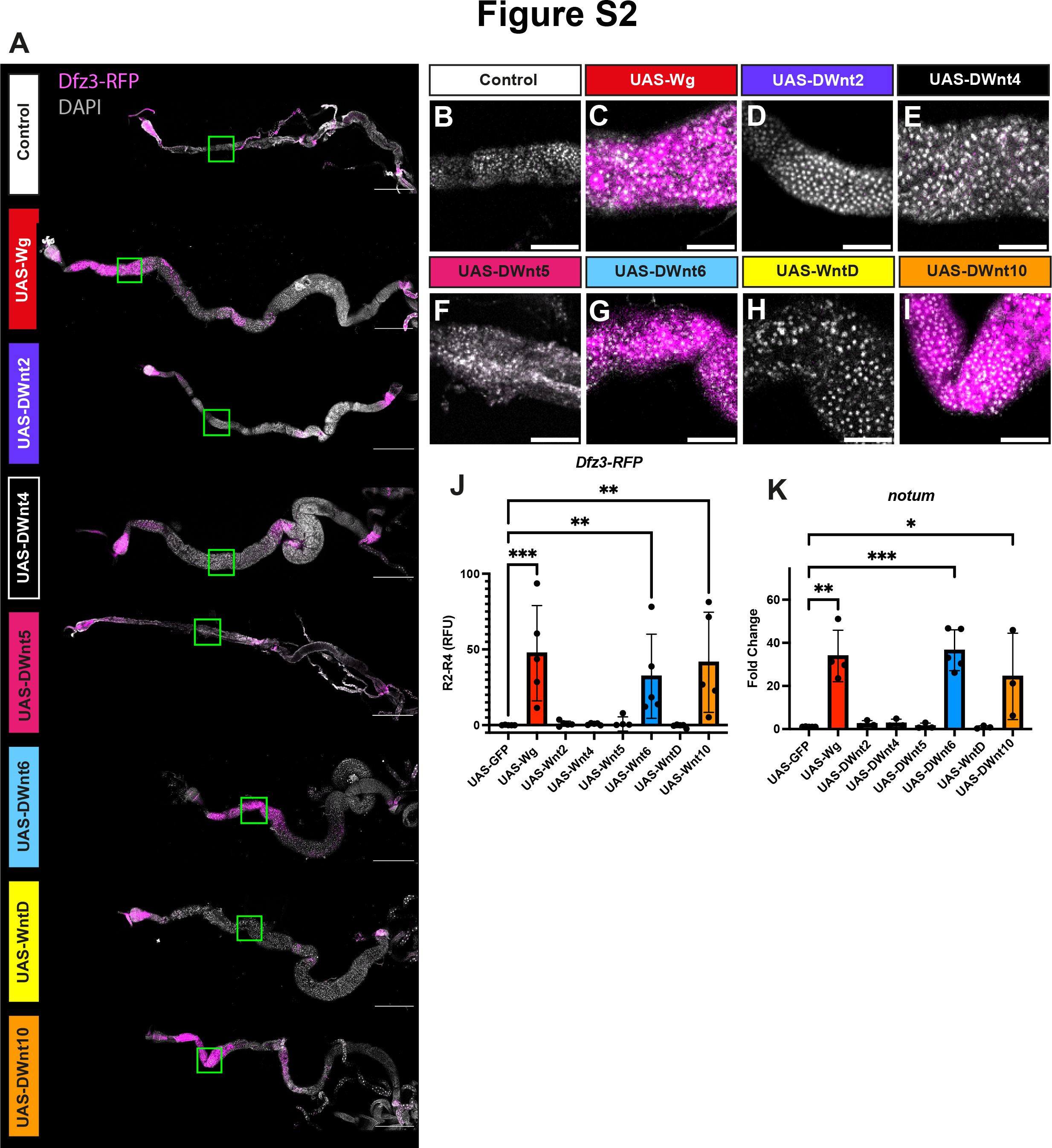


***Fig S2. Specific Wnt ligands trigger canonical pathway activation in adult guts 24 hours after induction.***

(A)Tiled confocal images (10×) of adult guts 24 h after induction of the *UAS/Gal4* system at 29°C (*tub-Gal4, tub-Gal80ts*), expressing the indicated *UAS-transgenes*. Guts were stained with DAPI (gray) and the canonical Wnt reporter *Dfz3-RFP* (magenta). Scale bars: 500 µm. (B–I) Higher-magnification confocal images of the R2 anterior gut region (green box in A). Scale bars: 100 µm. (J) Quantification of nuclear *Dfz3-*RFP intensity (relative fluorescent units, RFU) in R2 after background subtraction from R4 using FIJI. (K) qPCR analysis of the canonical Wnt target gene *notum* across all Wnt overexpression conditions. Bars represent mean ± SD. Statistical significance was determined using the Kruskal–Wallis test (****p < 0.0001, ***p < 0.001, **p < 0.01, *p < 0.05)
